# The use of complex clinical trials: a regulatory review

**DOI:** 10.1186/s13063-026-09674-8

**Published:** 2026-04-09

**Authors:** Quynh Nguyen, Hue Kästel, Katharina Hees, Benjamin Hofner

**Affiliations:** 1 Section Data Science and Methods, Paul-Ehrlich-Institut, Langen, Germany; 2 Department of Medical Informatics, Biometry, and Epidemiology, Friedrich-Alexander-Universität Erlangen-Nürnberg, Erlangen, Germany

**Keywords:** Complex clinical trial, Scientific advice, Systematic review

## Abstract

**Background:**

Complex clinical trials offer flexibility in evaluating multiple treatments or diseases simultaneously. These trials often feature adaptive designs, common controls, and the potential to add new arms. However, the increased complexity raises methodological challenges particularly regarding multiple testing or the adequate choice of control groups. Despite guidance from regulatory bodies like the European Medicines Agency and the U.S. Food and Drug Administration, uncertainties remain about the regulatory acceptance of these trials.

**Methods:**

This systematic review examines scientific advice procedures of products in the remit of the Paul-Ehrlich-Institut for complex clinical trials to highlight key concerns and regulatory feedback. We identified 30 scientific advice procedures corresponding to 29 different complex clinical trials.

**Results:**

Our findings reveal an increasing number of complex trial designs proposed by applicants. A lack of multiplicity control due to multiple arms was generally considered acceptable by regulators in exploratory trials and if cohorts can be considered independent. Additionally, the use of common control groups is frequently proposed by applicants. The review underscores the importance of pre-planning for new treatment arms, appropriate multiplicity control, and the definition of control groups in trial designs.

**Conclusion:**

Overall, our findings suggest that regulatory concerns regarding complex trials largely align with those in traditional trial designs, though their complexity requires careful, case-by-case consideration. Early engagement with regulatory agencies can be crucial to ensure the successful design and implementation of these trials.

**Supplementary Information:**

The online version contains supplementary material available at 10.1186/s13063-026-09674-8.

## Background

Complex clinical trials involve a broad range of trials with multiple (treatment) arms that share a common trial infrastructure such as the same master protocol and involve complex or adaptive trial features. The definition of these trial types can differ in the literature [6, 7, 37]. EU-PEARL has made an attempt to standardise relevant terms for complex clinical trials [8]. Commonly, they can be specifically classified further into basket, umbrella, matrix and platform trials. These trials have (complex) trial features such as the evaluation of multiple indications (i.e. different disease populations, or different tumour locations) for one treatment in one single trial (basket trial), the evaluation of multiple treatments for one indication in one single trial (umbrella trial), or a combination of both (matrix trial) [8]. In platform trials, new arms can be added to an ongoing trial which can potentially be perpetual, i.e. additional arms are added to an ongoing trial once they are available for evaluation with no pre-specified maximum number of arms in the trial [8]. Furthermore, common controls can be used in complex clinical trials, which can result in time-lagged control cohorts. These control groups can be categorised as either concurrent controls (e.g. control patients that are randomised at the same time as the respective treatment arm), or non-concurrent control groups (e.g. control patients that were randomised prior to an arm joining the trial). Adaptive design features can also be included such as interim analyses to stop arms due to futility or efficacy.

The flexibility inherent in complex clinical trials can enhance trial efficiency but also raises concerns about potential risks to trial integrity and data quality, as well as increased operational complexity [36]. The benefits of complex clinical trials are that multiple arms can be evaluated at the same time, potentially sharing a control arm (i.e. saving individual control patients) and using the same infrastructure [26, 27, 32]. This may reduce the time and number of patients needed to identify effective treatments [27]. Nevertheless, the increased flexibility also raises concerns regarding the need for multiplicity adjustment when evaluating multiple arms, how to implement interim analyses when adding or dropping arms, whether or not non-concurrent controls can be used and many more [6, 36]. Other than methodological issues, operational challenges e.g. for data management, or intervention supplies can also be increased [32]. Submission of complex clinical trials for clinical trial application require further considerations e.g. whether to submit a complex clinical trial under one or multiple clinical trial numbers [6].

Multiple regulatory guidance documents have been issued in recent years in Europe and the USA to guide the design and implementation of complex clinical trials. In Nguyen et al. [30], a detailed summary of relevant guidelines on topics such as multiplicity, the use of non-concurrent controls, allocation to treatment arms and simulations is provided. In addition, the relevant guidelines include information on operational and regulatory aspects such as the submission of clinical trial applications and marketing authorisation applications. Although these guidelines provide a good basis for e.g. designing and implementing complex clinical trials, not all aspects and issues have been addressed and need further considerations. Existing guidelines such as the guideline on multiplicity [10], adaptive designs [9, 35], or on the choice of the control arm [24] can be of relevance when complex clinical trial specific guidance do not yet cover these aspects. For example, Bofill Roig et al. [4] identified only four guidelines covering the use of non-concurrent controls (of which only one guideline provides guidance, which was against the use of non-concurrent controls due to potential time drifts). In contrast, 37 further guidelines provide information on the use of external or historical controls. It is apparent that non-concurrent controls share similarities to historical or external controls. This lead the authors to discuss the relevance and transferability to non-concurrent controls concluding that the requirements and concerns on the use of external controls are related to the use of non-concurrent controls but cannot be fully applied. On the one hand, usual concerns raised in the context of the use of external controls, such as selection bias or quality of the external control data, are lower for non-concurrent controls. But in return, additional sources of bias might arise when e.g. new treatments enter the trial or intermediate results are published, which may attract different patients than before. Regarding multiplicity, the US Food and Drug Administration (FDA) [36] generally does not recommend to adjust for multiplicity in umbrella trials as these trials can be considered similar to multiple independent clinical trials. Nevertheless, when using multiple doses, administration or formulations of the same drug, an adjustment is needed. In European guidelines [6, 11] multiplicity issues are mentioned as aspects to be addressed but no detailed guidance is provided so far. However, Nguyen et al. [30] observed that the majority of discussions in existing guidelines tend to focus on scenarios where multiple concurrent arms are in place. Scenarios where arms overlap only partially or not at all are not well covered at present.

At an ACT EU Workshop [13], it was noted that uncertainties regarding regulatory acceptability of platform trials exists, which slows down protocol design and adoption of platform trials. This may be attributed to the limited experience on both the developers’ and regulators’ sides, which restricts the exchange of both positive and negative insights. In the literature, experiences from developers have been shared for example by the study teams of ISPY-2 [2] or ALIC4E [5]. However, to date, there is limited literature on regulatory experiences, beyond the existing guidelines.

Therefore, we conducted a systematic text review on complex clinical trials in European regulatory documents with the aim of providing greater insight into the regulatory thinking, potential concerns or agreements from regulators, and how issues could be addressed by applicants. In comparison to existing literature reviews, we not only extracted trial characteristics such as the phase of the study, the indication(s), the number of treatment arms or the type of control from both running and planned trials but focused on the corresponding regulatory opinion on aspects specific for complex clinical trials. These aspects include for example the addition of new arms to an ongoing trial, the need for multiplicity adjustment, or the kind of control group proposed (e.g. common versus individual controls, or concurrent versus non-concurrent controls). As database, we used scientific advice procedures submitted to the European Medicines Agency (EMA). In a scientific advice procedure, developers have the opportunity to ask for guidance and directions from EMA on methods and trial design in a written format. Applicants provide questions and possible solutions. These are reviewed by regulators and a written scientific advice is given by the Committee for Human Medicinal Products (CHMP) via the Scientific Advice Working Party (SAWP). If needed, opinions from further experts, from other working parties or a discussion meeting with the applicant can be included in the scientific advice procedure. For further details regarding the scientific advice procedure and process flow, we refer to the official EMA websites [14, 15]. While naturally, pharmaceutical companies seek scientific advice, any developer is open to seek advice (e.g. academic institutions or other organisations). For simplicity, we will use the term applicant to refer to a developer or sponsor in a scientific advice and refer to CHMP’s and SAWP’s opinion as regulatory opinion. Since scientific advice procedures are confidential, we will not present individual trials or advice procedures but only present the results in summary or anonymised.

In the following section, we will outline our methodology for identifying scientific advice procedures related to complex clinical trials, along with the steps taken to extract relevant data. This will be followed by our results, which provide general information on the scientific advice procedures and the proposed clinical trials, including details on treatment arms, the addition of new arms, multiplicity control, and the type of control groups. We will then present three anonymised case studies that highlight common elements in complex clinical trials and the resulting scientific advice from the regulators. Lastly, we conclude with a discussion.

## Methods

To evaluate the use of complex clinical trials in scientific advice procedures, a systematic document review was conducted on documents that were submitted during a scientific advice procedure to the EMA with a data cutoff 2024-09-13. The systematic document review was performed according to the PRISMA 2020 guideline [31]. We only considered advice procedures on products which are in the remit of the Paul-Ehrlich-Institut (PEI) such as monoclonal antibodies, vaccines, and advanced therapy medicinal products. As complex clinical trials naturally consist of multiple arms, some trials may include arms or combination of treatments consisting of products in the remit of the PEI but also products in the remit of the Federal Institue for Drugs and Medical Devices (Bundesinstitut für Arzneimittel und Medizinprodukte, BfArM) [18] such as chemicals. A full list of products in the remit of the PEI and BfArM can be found at [33] and in section 77 of the German Medicinal Products Act [19]. The responsibility for an advice on multiple products primarily depends on the applicant who selects the primary type of product for the scientific advice procedure.

The database at PEI consists of more than 50,000 documents since the early 2000s covering around 2500 scientific advice procedures that were submitted to the EMA for review. The large amount of documents in contrast to the number of scientific advice procedures can be mainly explained by two aspects. First, documents that are submitted by applicants during a scientific advice procedure contain the briefing book, the trial protocol and can also include literature references, supporting protocols, reports and many other documents. Second, the database at PEI is also used as working environment resulting in multiple draft versions of the scientific advice in the database in addition to the final version. Furthermore, it also contains documentation of internal discussions and other internal documentation.

To reduce the amount of documents for our screening, we identified documents that might contain relevant information for the systematic review such as the trial protocol, the briefing book or the final advice letter in a programmatic way. Documents with a format other than.pdf or.docx were not accessible to the algorithm and thus omitted. The relevant keywords such as “protocol” or “briefing” (book) to identify the documents are provided in the Appendix, Table 6. Subsequently, we screened these documents using an R script (R version 4.2.3, [34]) for keywords related to complex clinical trials. Examples are basket, umbrella and platform trials, but also flexible trials, or multi-arm, multi-stage. A complete list of keywords is provided in the Appendix, Table 7. A manual screening of the resulting documents was performed to exclude documents that did not match our defined eligibility criteria (PICOS: Population, Intervention, Comparator, Outcomes, Study design). For instance, documents were excluded if a keyword appeared only in the reference list or if the term was used in a context unrelated to our context. Details of the PICOS are provided in the Appendix, Table 8. During the extraction, multiple documents were excluded such as draft versions of a scientific advice letter where the final advice letter was available. Additionally, many documents did not contain any of our pre-defined keywords related to complex clinical trials; however, as the corresponding scientific advice procedure had already been identified as related to a complex clinical trial, all relevant documents from the same procedure were retained for further extraction. A flow chart of each step is provided in the Appendix, Fig. 5. We extracted general information on the scientific advice procedures, such as the date, the number of questions and the scope of questions. Furthermore, we documented whether the trial was considered a basket, umbrella, matrix or platform trial by the applicant or by regulators. Among the operational features extracted were the number of indications, treatment arms, control arms, the randomisation and blinding as well as the sample size. In addition we obtained information on the planned analyses, interim analyses and multiplicity control. For each of these trial features, we tried to obtain the regulatory assessment and comments. The standardised extraction sheet can be found in the supplemental material.

The data was extracted by two independent reviewers QN and HK and adjudicated afterwards. In case of discrepant extraction, discussion was sought to solve the discrepancies, with the final consolidated dataset produced by the first reviewer (QN). No independent third reviewer was involved. The extracted information was analysed descriptively. The number of scientific advice procedures or trials in each pre-specified category was determined and free-text fields were summarised in listings.

## Results

### Scientific advice procedures

In total, we extracted information from 168 documents representing 30 scientific advice procedures and 29 different clinical trials. Documents included in a scientific advice procedure were for example first reports, briefing books, trial protocols or company presentations. For two trials, separate scientific advice procedures were sought, while for one advice, two trials were presented concurrently.

**Fig. 1:**
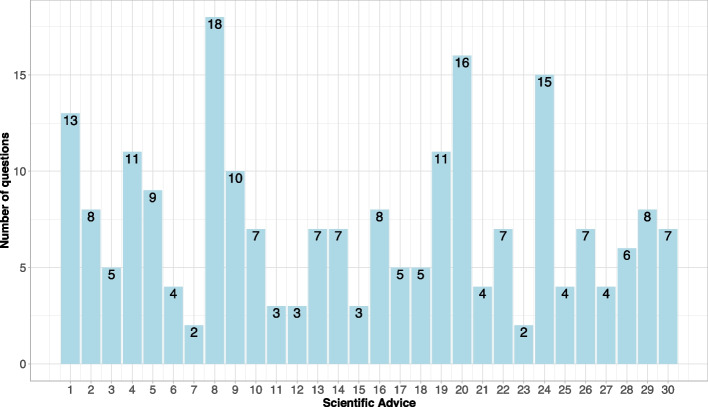
Overview of number of questions per scientific advice

**Fig. 2:**
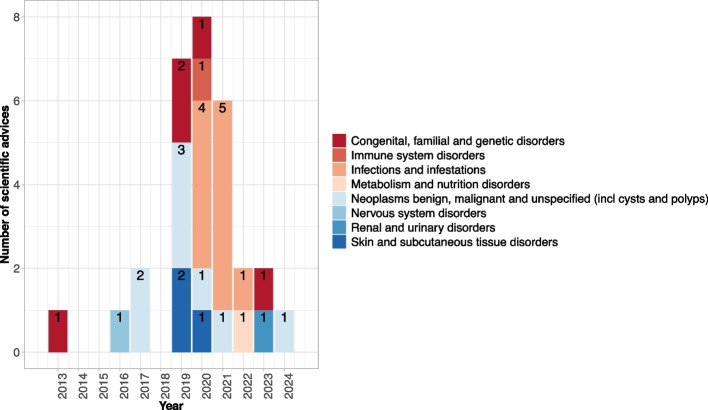
Trend of scientific advice procedures on complex clinical trials by disease groups over time

The number of questions included in a scientific advice varied from 2 to 18 (mean 7.3). This is only partially correlated with the complexity of the advice as no requirements on the number and content of questions exist. While some applicants separate each trial aspect into individual questions (e.g. on inclusion and exclusion criteria, on the endpoints, on the statistical analysis), others combine aspects into one overarching questions (e.g. on the clinical trial design including (a) inclusion/exclusion criteria, (b) endpoints, and (c) statistical analyses). The number of questions in each advice is depicted in Fig. 1. All advice procedures contained questions on clinical aspects, 18 additionally on non-clinical and 11 on quality aspects (multiple aspects per advice are possible). In 8 advice procedures, a discussion meeting was proposed by the regulatory assessment teams. Figure 2 shows the number of scientific advice procedures over time by different disease groups as defined by MedDRA primary system organ class (SOC). There has been an increase in the number of complex clinical trials over the years, with the highest number of scientific advice submissions in 2020 and the majority being proposed in the last 5 years. A wide range of disease groups is represented with the majority of trials in oncology (SOC: Neoplasms benign, malignant and unspecified), such as colorectal, renal, ovarian or breast cancer and lymphoma. In addition, many complex clinical trials have been proposed in COVID-19 (SOC: Infections and infestations) from 2020 onwards.

### General trial design of complex clinical trials

Since the definitions for complex clinical trials can vary in the literature [6, 8], we have defined these trial designs as follows for our text extraction:

- Basket trial: Investigate one treatment in multiple indications/diseases or disease subtypes
- Umbrella trial: Investigate multiple treatments in one indication/disease
- Matrix trial: Investigate multiple treatments in multiple indications/diseases or disease subtypes
- Platform trial: Basket, umbrella or matrix trial with flexible addition and dropping of arms or subtrials possible

**Fig. 3:**
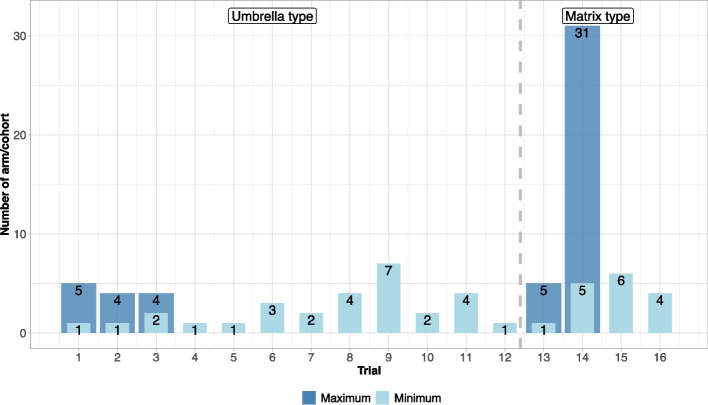
Number of (active) arms or cohorts in flexible trials, i.e. additional arms are allowed to be added to an ongoing trial (here only umbrella or matrix trials). Trials without a maximum number of arms represent potential perpetual trials as no maximum number of arms was proposed by applicants

With these definitions, each trial can only be categorised to be a basket, umbrella or matrix trial but no overlap of categories is foreseen. Furthermore, if a basket, umbrella or matrix trial has the possibility to add and drop arms during an ongoing trial, it is classified as a basket/platform, umbrella/platform or matrix/platform trial. Importantly, a trial must allow for both adding and dropping of arms to be classified as a platform trial. A trial with only a futility or early efficacy analysis alone without the possibility to add new arms would not be classified as a platform trial within this manuscript but is a basket, umbrella or matrix trial with group-sequential design aspects. In addition to futility or early efficacy analyses, other possibilities for dropping of arms on platform trials exist. Examples are when treatments are completed on the platform (i.e. completion of recruitment and analysis results are available), when external data supports the closure of an arm (i.e. knowledge of insufficient efficacy from another trial), when safety concerns arise, or when recruitment issues develop [21]. We acknowledge, though, that there is no clear and unequivocal definition. Based on these definitions, we identified 8 basket, 16 umbrella and 5 matrix trials. In addition, 16 of these trials had the flexible possibility to add and drop arms during the trial and were thus in addition classified as platform trials, of which 12 trials were of umbrella type and 4 of matrix type (see Fig. 3). In Table 1, general aspects on the proposed trials are presented. All possible phases of trials were represented with 11 out of 29 trials being phase III and the majority were confirmatory (21/29). A trial was classified as “confirmatory” if the applicant planned to use the trial for a future marketing authorisation application as a pivotal trial. More than half of trials were only in the planning phase when the scientific advice was sought (19/29) and double blind (15/29) (i.e. participant and investigator/sponsor team are blinded). For 13 trials, fixed designs were planned (i.e. no additional arms were planned to be added) with a median number of arms of 4 (minimum: 2, maximum: 10). In 23 trials, at least one interim analysis was planned with the intention of an early efficacy analysis (11/23), a futility analysis (19/23) or an adaptation of the study (9/23). Study adaptations included sample size adjustments (*n* = 4), change of study population (*n* = 2), change of the control group (*n* = 1) or dose modifications (*n* = 2) (categories are not mutually exclusive). Trials were considered as perpetual (11/29) in this extraction if no maximum number of arms or cohorts was provided but additional arms were considered to be added in the course of the trial. More than half of the trials (17/29) planned to enrol a total $\le$ 500 patients. The median sample size was 366 patients (minimum: 18, maximum 12,000, standard deviation: 2647.027) per trial.

**Table 1:** Overview of selected trial characteristics

Trial details (*N* = 29)	*n* (%)
Phase of study	
I	1 (3.45)
I/II	3 (10.34)
II	6 (20.69)
II/III	4 (13.79)
III	11 (37.93)
I/II/III	2 (6.9)
Not mentioned	2 (6.9)
Total sample size	
$\le$ 100	2 (6.9)
> 100 – $\le$ 500	15 (51.72)
> 500 – $\le$ 1000	3 (10.34)
> 1000	9 (31.03)
Status of clinical study as of scientific advice	
Recruiting	8 (27.59)
Planned	19 (65.52)
Not mentioned	2 (6.9)
Type of study	
Exploratory	6 (20.69)
Confirmatory	21 (72.41)
Not mentioned	2 (6.9)
Randomisation	
Equal	11 (37.93)
Unequal	5 (17.24)
Equal and unequal (in different study parts)	3 (10.34)
Minimisation	1 (3.45)
No randomisation	7 (24.14)
Not mentioned	2 (6.9)
Blinding	
Open label	11 (37.93)
Double blind	15 (51.72)
Double blind and open label (in different study parts)	3 (10.34)
Use of at least one interim analysis	
Yes, purpose of interim analysis^a^:	23 (79.31)
Early efficacy analysis	11 (47.83)
Futility analysis	19 (82.61)
Adaptive modification of study	9 (39.13)
No	3 (10.34)
Not mentioned	3 (10.34)
Addition of new arm during ongoing trial possible	
Yes	16 (55.17)
No	13 (44.83)
Maximum number of arms pre-specified	
Yes	11 (37.93)
No	18 (62.07)

^a^Purpose of interim analysis: Denominator is the number of studies with an interim analysis, categories are not mutually exclusive

Figure 3 displays the minimum and maximum number of arms (excluding control arms) proposed by applicants for each flexible trial (i.e. additional arms were planned to be added to the trial, *n* = 16). Trials allowing the addition of new arms are of umbrella trial and matrix trial type. In none of the basket trials additional arms were planned. One trial has a potential of a maximum of 31 arms. This trial is a dose-finding trial in multiple indications which was planned to be amended to include further dose-escalation cohorts of additional combination of treatments leading to the high number of cohorts.

**Fig. 4:**
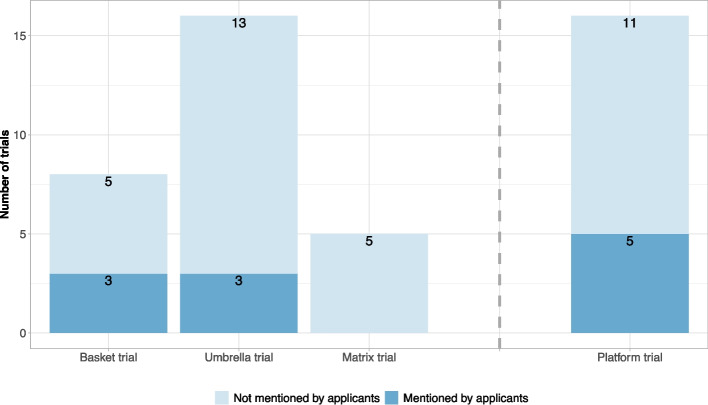
Classification of complex clinical trials into basket, umbrella, matrix and platform trial as mentioned by applicants

**Fig. 5:**
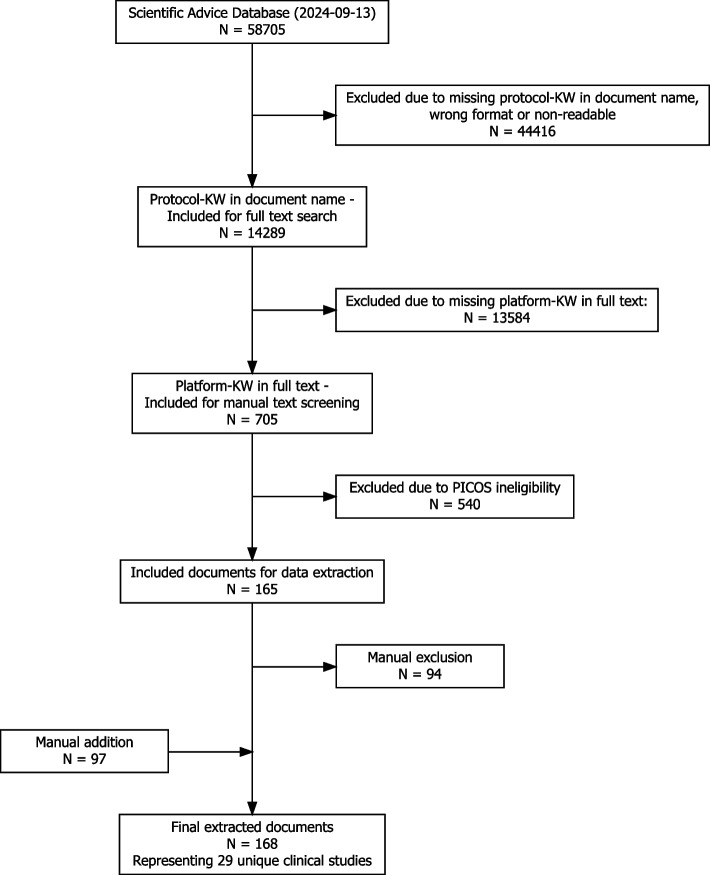
Flow diagram of scientific advice search. N: number of documents. Protocol-KW: keywords related to protocol, briefing book or list of questions. The number of documents for each keyword is provided in Table 6 in the Appendix. Platform-KW: keywords related to platform and complex clinical trials. A list of keywords and number of identified documents is presentend in Table 7 in the Appendix. PICOS: Following the PICOS (Population, Intervention, Comparator, Outcomes, Study design) criteria as defined in Table 8 in the Appendix, documents were included or excluded for data extraction and analysis

As depicted in Fig. 4, the majority of trials were not defined as basket, umbrella, matrix or platform trials by applicants within the scientific advice procedure. One reason for this might be the varying definitions of complex clinical trials in the literature. Especially matrix trials are not regularly defined in the literature. Usually, trials with both multiple treatment arms and indications are classified as either basket or umbrella trials. Another important aspect might be the relatively new emergence of these terms. While multi-arm trials with multiple treatments or indications have been planned and conducted in the past, the idea to label these trials with the terms basket, umbrella, matrix or platform trials has only recently been raised (see e.g. seminal papers by [3, 37]) and have gained more attention during the past years [27, 32]. Examples of early trials are the ISPY-2 [2], Battle [25], Lung-MAP [22] or NCI-MATCH [20] trials. Irrespective of the classification of these trials with specific terms, the use of multiple treatments or indications and potentially adding or dropping arms during a trial leads to more complexity compared to classical clinical trials. Thus, naturally concerns or conditions by regulators were raised during a scientific advice procedure. Among these were the following main concerns: the issue of multiplicity, the flexibility of opening and closing arms and its impact on the trial, the impact of the purpose of the trial (confirmatory vs exploratory), which arms are to be tested against which other arms, and the inter-dependency of arms (overlapping vs mutually exclusive arms). A short summary of regulatory advice for key aspects is provided in Table 2. A detailed elaboration and summary will be provided in the upcoming sections.

**Table 2:** Overview of key aspects in complex clinical trials and corresponding regulatory advice

Key issue	Regulatory feedback
Opening of (treatment) arms	• Justify the selected treatment and dose
	• Provide an appropriate definition of the resulting control group
	• Provide method for the incorporation of new arms
	• Provide considerations on the type I error control
Multiplicity control for multiple arms	• Justify the need or lack of multiplicity adjustments
	• Multiplicity control most likely needed in case of dependent arms
	• In case of multiplicity control: if possible show multiplicity control analytically
	• In case of no multiplicity control: Provide justification for independent arms
	– Basket trials: independent disease arms
	– Umbrella trials: independent therapies (e.g., targeting different biomarkers)
	– Decisions for each arm must be made independent of results for other arms
Control group	• Justify the choice of the control group
	• Preferably use concurrent control patients
	• Common controls are acceptable

### Opening of (treatment) arms

Opening of new arms in an ongoing trial under one protocol will usually be accompanied by a protocol modification such as an addition to an existing protocol via an appendix or a subprotocol which includes further details on the new arm. These modifications are generally submitted to authorities as a substantial modification or substantial amendment. Furthermore, a justification for the addition of the new arms must be submitted [11, 36]. In that case, regardless of what was prespecified in the (master-)protocol.

As mentioned above, 16 trials allowed the addition of new arms to an ongoing trial classifying them as a platform trial. All these trials were either of umbrella (*n* = 12) or matrix type (*n* = 4). Table 3 provides further details on the applicants’ proposal on timing and mechanism for adding new arms to an ongoing trial.

**Table 3:** Details on the timing and mechanism of addition of new arms or cohorts

Addition of new arm during ongoing trial possible (*N* = 16)	*n* (%)
Timing of additional arm	
After RP2D is established by the Sponsor	1 (6.25)
After phase I	2 (12.5)
After recruitment to the previous arms closed	1 (6.25)
After review by committee	3 (18.75)
After treatment becomes available for clinical testing	1 (6.25)
No details provided	8 (50)
Mechanism for adding new arm	
Appendix to the protocol	2 (12.5)
Based on emerging data	1 (6.25)
Protocol amendment	5 (31.25)
Subprotocol	1 (6.25)
No details provided	7 (43.75)

*RP2D*, recommended phase 2 dose

Categories for the timing and mechanism of adding new arms to a trial are somewhat overlapping. Generally, no specific details were presented by applicants but it is apparent that adding new arms was usually accompanied by a protocol amendment (*n* = 5), an addition of a subprotocol (*n* = 1) or an appendix to a protocol (*n* = 2). This is in line with current regulatory guidance for complex clinical trials [6, 36]. For some trials, a committee was planned to be implemented to decide on adding new arms. Importantly, regulators have commented that the addition of new arms must be thoroughly pre-planned and documented. A justification of the selected treatment and dose is needed at the time of the amendment. The majority of comments from regulators were related to the appropriate selection of the new treatment in a particular population. The documentation and justification should also cover an appropriate definition of the controls (i.e. which control patients are planned to be used), appropriate methods for the incorporation of new arms (e.g. when and how new arms are planned to be added), and considerations on the type I error control.

### Multiplicity control for multiple arms

All complex clinical trials discussed here are multiarm trials investigating multiple treatments or indications in one single clinical trial. Hence, the question on multiplicity control arises. Many aspects determine whether multiplicity control might be required or relevant in specific settings. While the scientific literature presents extensive arguments both in favour of and against multiplicity control, we furthermore examined regulators’ positions and acceptability of these arguments. Most commonly, the use of a common control group raises the discussion of multiplicity control as it leads to dependent test statistics. While the probability of at least one false positive finding (i.e. the familywise error rate, FWER) is smaller with a common control, the probability of at least two or more false positive findings is higher in comparison to using individual control groups [1, 23, 29]. As the FWER is not higher than using individual controls (whereby the latter can be seen as running individual trials for each active arm from a type 1 error perspective) many authors argue against the need for multiplicity control solely due to the use of a common control group. Additionally, the expected number of false positive findings is the same [1, 36]. Rather, it is argued, one should consider the potential dependency due to similar treatments or indications. Furthermore, it is elaborated in the literature that special trial situations such as orphan or rare diseases, or exploratory trials should be acknowledged when deciding on the multiplicity control [7, 23, 29].

In the scientific advice procedures, we extracted information on applicants’ proposals for and against multiplicity adjustments in complex clinical trials when using multiple arms. Other multiplicity-related adjustments, such as those required for interim analyses or the evaluation of multiple endpoints per arm, are beyond the scope of this review, as standard guidelines apply in those cases [9, 10]. Out of 29 trials, only 5 trials were proposed with multiplicity control due to multiple arms. For the remaining 24 trials, either no multiplicity control was foreseen (19/24) or no information on the multiplicity control was given by the applicants (5/24). The decision on the control for multiplicity by applicants and the corresponding regulatory feedback is often dependent on multiple factors. Table 4 displays the applicants’ proposals on multiplicity control for the 29 trials and the corresponding regulatory feedback by the type of complex clinical trial and whether the trials were planned to be confirmatory or exploratory.

**Table 4:** Applicants’ proposal on multiplicity control and regulatory feedback by type of complex clinical trial and confirmatory or exploratory purpose of the trial

Applicants proposal	T1E control (*n* = 5)	No T1E control (*n* = 24) ^a^
Regulatory feedback	Accepted/accepted with comments/concerns or conditions raised | not commented ^b^	Accepted/accepted with comments/concerns or conditions raised | not commented ^b^
Basket trial (*n* = 8)		
Confirmatory	n.a.	2/3/0 | 3
Umbrella trial (*n* = 16)		
Confirmatory	2/2/0 | 0	0/1/3 | 4
Exploratory	n.a.	0/0/1 | 3
Matrix trial (*n* = 5)		
Confirmatory	1/0/0 | 0	n.a.
Exploratory	n.a.	0/0/0 | 4

Numbers denote the number of studies in respective categories

* n.a.*, not applicable

^a^Includes 5 cases where no information on the multiplicity control was provided by applicants

^b^Accepted: Cases where regulators expressed acceptance without any further comments specific to complex clinical trials

Accepted with comments: Cases where regulators expressed acceptance but mentioned additional comments, concerns or conditions specific to complex clinical trials

Concerns or conditions raised: Cases where regulators did not express acceptance but raise concerns or conditions specific to complex clinical trials

Not commented: Cases where regulators did not provide comments on the relevant aspects specific to complex clinical trials

Multiplicity control was proposed by applicants in four umbrella trials and one matrix trial, all of which were designed as confirmatory studies. Reasons for multiplicity control were the use of a shared control (*n* = 3), the investigation of similar treatment arms (*n* = 3) or a single claim of efficacy (*n* = 3) (multiple reasons were possible). Proposed methods for multiplicity adjustments were closed testing procedure (*n* = 2), Bonferroni adjustment (*n* = 1), the Dunnett’s test (*n* = 1) and hierarchical testing (*n* = 1). Notably, the regulatory authorities endorsed the applicants’ multiplicity control strategies in all five cases. Nevertheless, for two trials, comments were made by regulators stating that the type I error control should not only be shown via simulation but an analytical solution was preferred.

Main reasons brought forward by the applicant against multiplicity control were that each arm would lead to an independent claim of efficacy (*n* = 11). These were basket and umbrella trials. In 5 of these cases, it was additionally argued that the platform was merely used for logistical reasons. In all these cases, the trials were planned as confirmatory trials. Other reasons were that at most one confirmatory test was planned in the multi-arm trial while the other arms were planned to be exploratory (*n* = 2) or that the trial was only an exploratory trial (*n* = 3). In eight trials, including five where the applicant did not specify whether multiplicity control was intended, no rationale was provided for the absence of multiplicity control.

For the basket trials, the proposal to treat each disease arm independently and therefore not to control for multiplicity control across arms was considered acceptable by regulators in most cases (see Table 4). It was noted that lack of type error control is only acceptable, if the cohorts are independent, non-overlapping, clearly defined and decisions for each cohort are made independent of the results of the other cohorts.

In confirmatory umbrella trials, a lack of multiplicity control was considered critical and concerns were raised by regulators. They declared, that the risk for inflated false-positive rates, arising from multiple testing, need to be addressed and a justification for the independence of arms would be required. In one umbrella trial that was planned to be exploratory, regulators stated, the claim that arms are independent would be accepted if e.g. therapies target different biomarkers. However, according to the given scientific advice, independence cannot be assumed in the case of same mode of action, different doses of the same therapy or combinations of already investigated therapies.

For matrix trials that were planned to be exploratory, no major comments were made by the regulators.

These findings align with existing guidance from the US FDA [36] where generally multiplicity adjustments are not needed if the “comparisons of different drugs to the control are aligned with distinct clinical objectives that would typically be evaluated in multiple independent clinical trials” [36]. However, exceptions are made when products are closely related such as multiple doses of the same drug. Guidance from the EMA is still pending (see Concept paper; [12]).

### Control group

Control groups in complex clinical trials can vary in many ways. We have identified two dimensions to describe a control group in complex clinical trials: (1) the general type of control group, and (2) the concurrency of the control group.

For the first dimension, the following possible control groups are considered from our search in the scientific advice procedures: individual controls per arm, one common control for all arms, individual controls per arm which are merged into one common control for analysis, external controls or no control at all. In practice, other possible control group settings may be available such as common controls for subgroups of active arms, leading to more than one shared control arm in the trial.

For the second dimension, we distinguish between concurrent controls (i.e. patients randomised at the same time as the experimental arm) and non-concurrent controls (i.e. patients randomised to the control group before the experimental arm joins the trial). While the use of a common control group is generally accepted by the FDA [36], the use of non-concurrent controls, resulting from new arms joining the trial, is discouraged due to the potential for bias. Nevertheless, the FDA guidance [36] discusses circumstances where the use of non-concurrent controls may be justified. Similarly, in the European guideline [6], the use of concurrent controls is preferred if there are changes over time and a discussion on the impact on the comparison to the control arm is recommended. Since non-concurrent controls share similarities to historical or external controls, existing guidelines such as the guideline on the choice of the control arm can be consulted [24]. Further guidelines covering the use of external control data were summarised by Bofill Roig et al. [4]. In practice, the definition of concurrent and non-concurrent controls may be further refined by including conditions on e.g. the eligibility of patients. Concurrent controls can then be defined as patients who are randomised at the same time as the experimental arm and meet the same eligibility criteria as the experimental arm.

In Table 5, we display the proposal by applicants of control groups in complex clinical clinical trials and the corresponding regulatory feedback. We only included comments, concerns or conditions from regulators if they were specific to complex clinical trials. General comments were not considered for the classification in Table 5. Notably, for the majority of the studies, no specific comments were raised by regulators concerning the proposed control group in a complex clinical trial. In most cases, comments were made merely concerning aspects of the choice of control groups which were not specific to complex trials but were of general nature (e.g. whether the chosen control group is adequate in a specific indication or setting). These were out of scope for Table 5. Nevertheless, we will present selected comments on the proposed control group which we consider interesting or specifically relevant for complex clinical trials.

**Table 5:** Applicants proposal for control groups and regulatory feedback by type of control and concurrency of controls

Type of trial	Proposed control groups	Accepted/accepted with comments/concerns or conditions raised/not accepted | not commented ^a^
Basket trial (*n* = 8)	Individual controls only	1/0/0/0 | 5
	No control	0/1/0/0 | 1
Umbrella trial (*n* = 16)	Common control only	
	Concurrent	0/3/0/0 | 2
	Individual controls merged to common control	
	Concurrent	0/0/0/0 | 4
	Concurrent and non-concurrent	0/0/1/0 | 0
	Individual controls only	1/0/0/0 | 1
	External control	0/0/0/1 | 0
	No control	0/1/0/0 | 2
Matrix trial (*n* = 5)	Common control only	
	Concurrent	0/0/0/0 | 1
	Individual controls merged to common control	
	Concurrent	0/0/0/0 | 1
	External control	1/0/0/0 | 0
	No control	0/0/1/0 | 1

Numbers denote the number of studies in respective categories. Empty combinations are suppressed in the table

^a^Accepted: Cases where regulators expressed acceptance without any further comments specific to complex clinical trials

Accepted with comments: Cases where regulators expressed acceptance but mentioned additional comments, concerns or conditions specific to complex clinical trials

Concerns or conditions raised: Cases where regulators did not express acceptance but raise concerns or conditions specific to complex clinical trials

Not accepted: Cases where regulators expressed disagreement without any further comments specific to complex clinical trials

Not commented: Cases where regulators did not provide comments on the relevant aspects specific to complex clinical trials

In 12 trials, common controls or individual controls merged to a common control for analysis were proposed by applicants. All these trials where of an umbrella or matrix type. The use of common and concurrent controls (*n* = 6) was generally accepted by regulators with the following remark: some trials use a placebo control group which may need to be revised once active treatments in the target population are approved and available. This is of particular importance for platform trials due to their adaptive nature with arms entering and leaving, leading to a longer overall trial duration.

In 5 other trials, individual concurrent controls merged to a common control for analysis were proposed. Concerns from regulators for these trials were mainly on the choice of the control treatment (e.g. a different control treatment was preferred by regulators) but none on the merging of control groups.

For 1 trial, the use of both concurrent and non-concurrent controls was introduced by applicants. Regulators raised two concerns. First, merging of control patients can only be seen uncontroversial if the patients in the control groups appear to be similar. Differences in patient management or outcomes will result in challenging interpretation. Second, comparison to non-concurrent controls adds a another layer of complexity and a comparison to the individual (concurrent) control group is preferred.

For 2 trials, external controls were proposed. In one case, a robust external control group was in principle considered acceptable by regulators in the given situation of a phase I/II trial. In the other case, the use of an external control was not considered acceptable in a confirmatory phase II trial. The applicant argued that a randomised trial with an active control group would not be feasible. Though not specific to complex clinical trials, regulators did not consider it sufficiently well justified and stated that they prefer a small randomised trial to a single arm trial.

Lastly, in 7 cases, uncontrolled clinical trials were proposed by the applicants. For 3 trials, comments were made by regulators: in one trial, a basket trial, the absence of a control group was considered acceptable since within-subject comparisons were foreseen. However, such a comparison could lead to bias and therefore regulators requested a discussion of potential sources of bias and the expected impact on the treatment effect estimates in the trial protocol. In the second case, an umbrella/platform trial in a rare disease was proposed. It was acknowledged by the regulators that in this case a common control would be heterogeneous and might limit the interpretability of results. Hence, the absence of a comparator arm was considered acceptable. The last trial was a phase I trial and the lack of a control group was considered acceptable by the regulators. However, a comparison to an active control group is expected in future trials.

## Case studies

In the following, we present 3 examples of trials proposed by applicants in scientific advice procedures and the corresponding advice or comments given by regulators. Selected aspects from the proposals and advices are presented and do not entirely represent the whole trial proposal, questions and advices. They were chosen as they were considered to further illustrate some of the aspects discussed above. These examples shall provide a better understanding for accepted aspects, thoughts and concerns from a regulatory point of view. Furthermore, examples were not chosen such that we necessarily endorse all comments or recommendations. They rather reflect the CHMP’s view at the time of the advice.

### Umbrella-type platform trial with a common control

This case study was proposed by the applicant as a flexible phase III, multi-arm, multi-stage design with the possibility to add or terminate trial arms due to futility or overwhelming efficacy. The trial was supposed to be started with three arms: (1) monotherapy, (2) combination therapy (combined with the monotherapy treatment) and (3) a common control group. Co-primary endpoints were planned for each comparison to the control group. Additional arms may be added based on emerging external data. The applicant argued that such a design allows faster evaluation of treatments than conducting single trials. The sample size calculation accounted for multiple comparisons using Dunnett’s test for multiplicity control across arms and hierarchical testing of the co-primary endpoints for multiplicity control within each arm. The applicant provided the estimated FWER for a 3-arm design (original proposal) and a 4-arm design (potential amendment to include a new arm) via simulation. In general, this was agreed by regulators but analytical confirmation of the type I error control would have been preferred. When adding a new treatment arm 4, the applicant confirmed that the comparison will only be conducted against concurrently randomised patients to the control group. This was welcomed by the regulators. Nevertheless, the regulators raised the concern that the introduction of an additional arm could result in a changed patient population, which could possibly affect the other arms: When adding a new treatment arm, the population before adding arm 4 and after adding arm 4 could differ leading to potential bias when pooling the data for each arm (i.e. patients from before adding arm 4 and after adding arm 4), and thus also their comparison to the control arm could be biased. This change was considered to go beyond natural drifts over a long recruitment phase as the addition of a new treatment arm may additionally influence the recruitment. The potential change in eligibility criteria, the change in the probability to be enrolled to a specific arm (including but not limited to the control arm), and the possibility to be enrolled to a new treatment arm (which is considered either especially promising or exhibits potentially severe side effects) may change the willingness of patients (and investigators) to join the trial. This could lead to increased differences in the patient population over time, thus hampering the analysis. The regulators therefore suggested to keep the treatment separate such that the new arm was only to be added once recruitment to the other arms was complete. Since no further details on the addition of a new arm was available, the applicant was advised to return for a follow-up advice.

### Umbrella-type platform trial without a control arm

The proposed study was a phase II, multi-arm, non-randomised trial with the possibility to add and drop treatment arms. Patients would be allocated to treatment arms depending on the biomarker profile of their tumour. If a patient was present with multiple biomarkers, the applicant proposed to assign the patient to the biomarker arm with the lower expected prevalence. No control group was foreseen. In the initial design, 3 treatment arms were defined each with a different set of mutations. In a 4th arm all patients without any mutations covered by the other 3 arms were enrolled. If new treatments become available, the applicant planned to add new interventions to the trial via a protocol amendment. While the applicant initially proposed the design as an exploratory trial and thus did not provide power and sample size calculations, a potential type II variation or marketing authorisation application filing depending on the results from the trial was mentioned by the applicant in a discussion meeting. The primary objective of the trial was to evaluate efficacy separately for each biomarker driven treatment arm. The applicant claimed that each treatment arm can be considered independent similarly to separate single-arm trials and therefore no multiplicity adjustment would be needed. This was partly acceptable for regulators with the following concern: independence can be agreed if the proposed treatments target different biomarkers. However, this is not necessarily the case if treatments have the same mode of action, or combinations of already investigated treatments are evaluated in further arms. Therefore, agreement could not be provided in general by the regulators as more information would be needed before other arms are added to the trial.

The applicant furthermore proposed the possibility for patients from discontinued treatment arms or those who experienced a disease progression on treatment arms to enrol in one of the other active arms. This was discouraged by regulators in a confirmatory setting as the baseline of these patients could differ and it would be difficult to determine to which arm a treatment effect would be allocated to. Regarding the 4th arm, regulators raised the concern that it is unclear how the definition of patients allocated to arm 4 (i.e. no mutation covered by the other arms) will change if one of the other arms is closed or new biomarker defined arms are opened. In general, the applicant was referred to the CTFG recommendation paper on the initiation and conduct of Complex Clinical Trials with regards to the addition of new arms [6].

### Basket trial with 3 indications and individual controls

This study was proposed as a pivotal trial in a group of orphan diseases which can be classified by different conditions caused by different genetic defects. These conditions share similar pathophysiological and clinical features. Due to the rare conditions, challenges were identified by the applicant, e.g. to identify a sufficient number of patients per disease. However, considering the shared pathophysiological features in the conditions, a single trial was nevertheless deemed feasible by the applicant and would allow for operational efficiency. The experimental treatment was the same for all cohorts and already approved in other indications. The applicant planned to use the trial as pivotal evidence for an approval of an indication extension. For each condition, a separate cohort was planned with the experimental treatment versus an individual placebo group. Regulators acknowledged the plan to include all three conditions in one protocol due to mainly logistical reasons. Nevertheless, the applicant was requested to provide further justification on the inclusion of the chosen conditions in one protocol as differences in the conditions or in recruitment rates were considered to be a potential issue. Each cohort was planned to be analysed separately without an overall success criterion defined across cohorts. Therefore, the applicant did not plan to adjust the type I error rate due to testing of multiple cohorts. Treating each cohort independently was acceptable for regulators and it was agreed that no type I error adjustment was necessary. An additional interim analysis for efficacy was proposed by the applicant. It was planned after data on the primary efficacy endpoint was available from approximately two thirds of the patients (across all three conditions). Early stopping would be based on pooled data from all cohorts. This proposal was questioned by regulators as pooling of all cohorts at the interim analysis would be in contrast to the previous argument against multiplicity adjustment and separate analyses of cohorts. Nevertheless, due to the similarity of the conditions, an interim analysis based on pooled data across the conditions may be considered acceptable by regulators. However, given the concerns regarding multiplicity, it was highlighted that this would need to be accompanied by evidence in the individual cohorts. Furthermore, the applicant was encouraged to ensure an even distribution of patient allocation to the different conditions.

## Implementation of regulatory feedback

Although it is not in the primary scope of our manuscript, it is of interest to see whether regulatory feedback had an impact on the final trial design. Therefore, we searched for the clinical trial protocols of the trials, for which the applicants requested scientific advice, on US and European trial registries [16, 17, 28], meaning that these protocols were submitted for clinical trial application (data cutoff: 2026-02-20). We reviewed key aspects discussed in this manuscript to provide a high-level overview of implementation or changes to the trial design after the scientific advice was provided.

Out of 29 clinical trials, 21 trials were initiated (11 thereof completed or prematurely terminated; 10 still ongoing). The type of complex trial was as follows: 7 basket trials, 10 umbrella trials and 4 matrix trials. In 14 of these trials, additional arms were allowed to be added during the ongoing trial, classifying them additionally as platform trials. For 8 trials, no protocol was submitted to any of the trial registries and the trials were also not mentioned on the applicant’s website. Potential reasons were presumably that the company does not exist anymore, a change in the development programme of an applicant, or that the compound did not complete the non-clinical research (yet).

The mean duration of trials (*N* = 21), defined as the time between the start of recruitment and date of trial completion, was 60.72 months (minimum: 5.16 months, maximum: 194.13 months, standard deviation: 42.84 months). For ongoing trials, the expected date of trial completion as specified by the applicant on the trial registries was used. The minimum corresponds to an early terminated trial, while the maximum represents an ongoing trial which did not specify the maximum number of treatment arms yet. The median number of treatment arms on the trial was 5 (minimum:2, maximum 27, standard deviation: 6.66). For ongoing trials, the current number of arms was used and may increase in the future. For completed platform trials the median number of additional arms was 4.5 (minimum: 1, maximum: 12, standard deviation: 4.31). The moderate number of additional arms in platform trials shows that platform trials mostly terminate after a reasonable number of additional arms.

Regarding multiple testing in the initiated confirmatory trials (*n* = 18), 15 trials did not control for multiplicity across all arms in the trial. The main rationales were that only one confirmatory test was planned (*n* = 5) and that independent inferential arms were used (*n* = 9). In the latter case, none of the applicants provided a sufficient justification for the choice of multiplicity control and did not justify the independence of arms specifically in the statistical methods section of the protocol. The rationale was only implicitly provided in the sections on the rationale for the trial or the rationale for the chosen treatments or indications by describing the different modes of action of treatments and/or indications. This is not fully in line with regulatory advice. In one case, regulators had expressed concerns in the scientific advice on the multiplicity when using multiple doses of the same drug without multiplicity adjustment. This was considered by the applicant and multiplicity adjustment was implemented.

All trials that had planned for a common, concurrent control group also proceeded with the plan. To ensure the appropriateness of the common controls or when individual control groups are merged to one common control group, some protocols further defined that the control group for each individual treatment arm was defined as the subset of common control patients that were also eligible for the corresponding treatment arm (i.e. ensuring the same inclusion and exclusion criteria for the treatment-control comparison). One trial planned to use concurrent and non-concurrent control patients during the scientific advice. Though regulators had raised concerns and preferred concurrent controls only, the applicant proceeded with the use of non-concurrent controls, and additionally defined a potential sub-protocol specific external matched-control group in the master protocol after the scientific advice was provided. As of now, none of the intervention specific sub-protocols of that master protocol described the need for an external matched-control group. Two other trials planned to use external controls during the scientific advice procedure, of which one was not conducted and in the other one the external control was omitted from the protocol after the scientific advice was provided.

## Discussion

Complex clinical trials offer great flexibility in studying multiple treatments or diseases with complex or adaptive trial features such as using common controls or adding new arms to an ongoing trial. With higher complexity, operational and methodological issues increases as well. Scientific literature on aspects such as randomisation and treatment allocation, multiplicity and non-concurrent controls is increasing. A number of regulatory guidances have been issued in the past years [6, 12, 36] with upcoming guidance expected such as the final version of FDA’s guideline on master protocols [36], or EMA’s reflection paper on platform trials following the concept paper [12]. Nevertheless, during an ACT EU Workshop [13], uncertainty regarding regulatory acceptance of platform trials was noted.

Therefore, we performed a systematic review in scientific advice procedures on complex clinical trials to provide further insights into relevant trial aspects corresponding comments or concerns from regulators. Our findings mostly align with the scientific literature. We observed an increasing number of such trials in recent years, a trend also identified in other systematic reviews [27, 32]. In general, applicants proposed complex clinical trials to improve efficiency such as using a shared logistical platform and common infrastructure. Therefore, mostly no multiplicity control across multiple arms was proposed by applicants. This lack of control was usually justified by the perceived independence of the trial arms. For example, in basket trials, the arms were justified as independent due to distinct patient populations, while in umbrella trials, independence was claimed based on the use of different treatments. In most cases, if sufficient justification was provided by the applicant, the argument for independence and hence the lack of multiplicity control across arms was usually acceptable for regulators. On the other hand, multiplicity control was most likely requested if dependencies between the arms existed. Examples of such dependent arms, which were mentioned by regulators in our review were trials containing treatments with the same mode of action, different doses of the same therapy or combinations of already investigated therapies. Discussions on the independence of arms are also present in the literature and align with our observations here [7, 23, 29].

In basket trials, we noticed that mostly individual controls were planned. In the umbrella trials, both common and individual controls were proposed. In some cases, the individual controls were planned to be merged to a common control for analysis. Main comments from regulators were that pooling of patients would only be acceptable if these patients appear to be similar. Differences in patient management or outcomes were provided by regulators as examples for dissimilarity.

With regards to opening new treatment arms, regulators requested that these would need to be pre-planned with justification of the selected new treatment, appropriate definition of the control arm and appropriate considerations on the type I error control. Notably, for basket trials, no additional (disease) arms were planned to be added during the ongoing trial.

Our findings on the addition of a new arm align with existing guidance from EMA and US FDA [12, 36], and findings on multiplicity control and the use of concurrent control patients align with the existing guidance from the US FDA [36]. Guidance from EMA on methodological aspects is currently pending but a reflection paper on platform trials following the concept paper [11] is in preparation. Nevertheless, it is overall noticeable, that in general, the comments and concerns from regulators are not specific to only complex clinical trials. These are concerns also seen in “classical” trials. They may appear more explicitly in a complex clinical trial due to the combination of multiple complex aspects and as multiple development programmes may share a single trial platform. For most aspects, regulatory guidelines are available and can be used to understand common concerns and expectations from regulators. Examples are guidelines on multiplicity [10], adaptive designs [9, 35], or on the choice of control arm [24].

In a second search, we identified the trials, for which the applicants requested scientific advice, in trial registries [16, 17, 28] to see whether the scientific advice had an impact on the initiated trials. Though the extraction was only high-level, we found that platform trials usually terminate after a reasonable number of additional arms. Statistical concerns, such as the change in patient population or change in standard of care over time, which may result in bias, are relevant and need to be considered. However, with a reasonable study duration in practice, these concerns may be reduced. The regulatory concern of everlasting trials was not reinforced either, at least in these trials. We noticed that multiplicity adjustment over treatment arms was often not implemented and a clear justification was missing in the protocol. A brief justification, potentially with cross-reference to the relevant clinical sections describing the treatment arms and study rationale is considered of great importance for a scientifically sound confirmatory trial. To address regulator’s concerns on the appropriateness of the common control group it was often defined that only subjects eligible for the corresponding treatment arm were to be considered.

### Strengths and limitations

Systematic reviews of published complex clinical trials have been performed in the past [21, 27, 32] summarising characteristics from complex clinical trials. As scientific advice procedures are confidential and usually not published, we provide a unique summary on the discussion of complex clinical trials between applicants and regulators. Our review complements the existing reviews of complex clinical trials with the current regulatory thinking and potential concerns or agreements from regulators at the time of the scientific advice request.

We acknowledge that our systematic review has several limitations. First, not all trial designs that are planned or conducted have a preceding scientific advice. Second, our database at the PEI does not include all EMA scientific advice procedures. In general, it contains mostly products in the remit of the PEI [33], while in fact these trials could also contain other products not in the remit of the PEI. Third, in some years, only scientific advice procedures where the PEI acted as coordinator were included. Hence, our search was potentially prone to selection bias. However, the resulting comments and concerns from regulators were mainly related to methodological aspects rather than being product specific. Thus, the recommendations and requirements should also be applicable to non-PEI products. Notably, the classification of complex trials into terms such as basket, umbrella, matrix or platform trials was not entirely aligned between applicants and the literature. Due to inconsistent definitions and non-existent definitions of complex clinical trials in the past, we likely have missed to identify trials with complex trial features that were not labelled as such. These factors explain the lower number of identified complex clinical trials in comparison to existing systematic literature reviews based on scientific publications. Nevertheless, the number of identified trials in our search can be considered a lower bound of trials and our results can serve as a first basis for common requirements or concerns from a regulatory point of view.

### Conclusion

With increasing experience of designing and implementing complex clinical trials, designs from complex clinical trials may change and differ from the trials identified in the review. Similarly comments and concerns from regulators may be subject to change with increasing exposure and learning from complex clinical trials. It is clear that each complex clinical trial is different and should be designed and evaluated case by case. Depending on many factors such as the objective of the trial, the indication, the available supporting information, the justification, and the therapeutic field, regulatory acceptance of specific aspects may differ. Thus, applicants are encouraged to seek scientific advice on complex clinical trials as early as possible.

## Supplementary information

Additional file 1. The standardized extraction sheet.

Additional file 2. The PRISMA 2020 Checklist.

## Data Availability

Not applicable. The datasets generated and analysed are not publicly available as scientific advice procedures are confidential.

## Appendix

**Table 6:** Protocol-KW: keywords related to protocols and briefing books

Keyword	Number of documents
protocol	904
briefing	1422
bb	490
bp	346
bd	175
briefdoc	27
Report	9046
jar	42
jr	207
list	835
loi	322
loq	13
minutes	662
clarification	241
question	171
response	388
Document with at least one search term	14289

Each keyword is case insensitive. Searches were performed for flexible start and end of each keyword (e.g. protocols was identified through protocol). *bb* briefing book, *bp* briefing package, *bd* briefing document, *jar* joint assessment report, *jr* joint report, *loi* list of issues, *loq* list of questions

**Table 7:** Platform-KW: keywords related to platform and complex trials

Keyword	Number of documents
subprotocol	21
sub-protocol	11
study_group_platform	2
adaptive_platform	31
umbrella_platform	5
umbrella_or_basket	1
basket_comparator	8
basket_of_clinical_comparator	1
multi-arm_multi-stage	7
multi-arm_,_multi-stage	12
intervention_specific_appendi	4
complex_clinical_trial	17
complex_stud	31
complex_trial	6
platform_trial	105
master_trial	5
umbrella_trial	48
basket_trial	45
matrix_trial	1
platform_clinical_trial	5
umbrella_clinical_trial	2
basket_clinical_trial	1
platform_stud	157
master_stud	6
umbrella_stud	83
basket_stud	174
matrix_stud	20
platform_protocol	6
master_protocol	128
umbrella_protocol	3
basket_protocol	13
platform_design	51
umbrella_design	26
basket_design	13
matrix_design	24
platform_randomised	3
MAMS	5
Document with at least one search term	705

Each keyword is case insensitive. Searches were performed for flexible start and end of each keyword. Underscore denotes space in the search

**Table 8:** PICOS (Population, Intervention, Comparator, Outcomes, Study design)

Category	Inclusion criteria
**P**opulation	Humans
**I**ntervention	No restrictions
**C**omparator	No restrictions
**O**utcomes	No restrictions
**S**tudy design	Complex clinical trials, including
	- Platform trials
	- Basket trials
	- Umbrella trials
	- Matrix trials
	- Master protocols
Other	Language: English
	Direct specification in the document (exclude indirect citation)
	Document type: Only actual protocols
	(e.g. exclude communications with term protocol in document name)

PICOS defines the inclusion and exclusion criteria for the documents eligible for manual text and information extraction

## Abbreviations

CHMP: Committee for Human Medicinal Products
CTCG: Clinical Trials Facilitation and Coordination Group
EMA: European Medicines Agency
FDA: Food and Drug Administration
FWER: Familywise error rate
n.a.: Not applicable
PEI: Paul-Ehrlich-Institut
PICOS: Population, Intervention, Comparator, Outcomes, Study design
RP2D: Recommended phase 2 dose
SAWP: Scientific Advice Working Party
SOC: System organ class
