# Supplementary material for: The use of complex clinical trials: a regulatory review
Source: Trials. 2026 Apr 9;27:289. doi: 10.1186/s13063-026-09674-8 (PMC13067453; doi:10.1186/s13063-026-09674-8)
Supplement: Supplementary file 1 — Additional file 1. The standardized extraction sheet. [file 13063_2026_9674_MOESM1_ESM.pdf]

									General document data			
									Drop-down	Free Text	Free Text	Date
Reviewer 1	Reviewer 2	Adjudicator	Path	Removal Flag	Removal Other	Main ID	Manual Addition	Study Name	Type of document	Type of document: Other, additional info	Version	Date of document

General study				Scientific Advice header							
Drop-down	Free Text	Drop-down	Free Text	Automatic extract	Automatic extract	Automatic extract	Automatic extract	Automatic extract	Free Text	Free Text	Automatic extract
Is a statistician involved?	Why is this study considered a complex study?	Phase of study	Other: Phase of study	Invented_name	Active_substance	Pharmaco_therapeutic_group	Indication	Application	Expected Plenary discussion	DM (discussion meeting) proposed	Eudra

Other automatic extracts		Scientific advice feature					Complex study feature				
		Scope of questions					Type of study				
Automatic extract	Automatic extract	Drop-down	Drop-down	Drop-down	Integer	Integer	Drop-down	Drop-down	Drop-down	Drop-down	Free Text
IND	NCT	Non-clinical	Clinical	Quality	Number of questions/issues	Number of questions/issues related to complex clinical trial design	Basket Study	Umbrella Study	Platform Study	Matrix study	Other

				Status of complex study					
Drop-down	Drop-down	Drop-down	Free Text	Drop-down	Free Text	Date	Date	Integer (Months)	Drop-down
Is a master protocol mentioned	Are subprotocols for each treatment arms mentioned?	Advice/Concern regarding complex clinical trial	Other/Comments: Advice regarding complex clinical trial	Status of complex study as of document submission	Other: Status as of document submission	Start date of recruitment	End date of recruitment	Planned trial duration per patient	Perpetual trial?

	General							
Drop-down	Free Text	Drop-down	Free Text	Integer	Integer	Integer	Integer	Drop-down
Advice/Concern regarding trial duration	Other/Comments: Advice regarding trial duration	Type of study	Other/Multiple: Type of study	Number of minimum unique investigational product	Number of maximum unique investigational product	Number of minimum indications/sub-populations	Number of maximum indications/sub-populations	Advice/Concern regarding products and population

	Treatment arms								
Free Text	Integer	Integer	Drop-down	Free Text	Free Text	Drop-down	Free Text	Drop-down	
Other/Comments: Advice regarding products and population	Planned number of minimum arms/cohorts	Planned number of maximum arms/cohorts	Addition of new arm/cohorts in ongoing trial possible?	Timing of additional new arm	Mechanism for adding new arm	Type of treatment arms/cohort	Type of treatment arms/cohorts: Other	Advice/Concern regarding treatment arms	

	Control arms								
Free Text	Drop-down	Free-Text	Integer	Drop-down	Drop-down	Drop-down	Free Text	Drop-down	Free Text
Other/Comments: Advice regarding treatment arms	Type of control	Other: Type of control	Number of control arms	Use of common control	Use of individual control groups	Advice/Concern regarding control	Other/Comments: Advice/Concern regarding control	Randomization scheme	Randomization scheme: Other

Randomization and blinding								
Free Text	Drop-down	Integer	Drop-down	Free Text	Drop-down	Free Text	Integer	Integer
Randomization Ratio	Stratification?	Number of stratification factors	Blinding	Other: Blinding	Advice/Concern regarding randomization/blinding	Other: Advice/Concern regarding randomization/blinding	Planned total sample size	Planned sample size reassessment?

Sample size								
Free Text	Free Text	Drop-down	Free Text	Drop-down	Free Text	Drop-down	Free Text	Drop-down
Planned sample size per treatment arm	Planend sample size per control arm	Sample size calculation	Other: sample size calculation	Advice/Concern regarding sample size	Other: Advice/Concern regarding sample size	Primary endpoint variable	Other/Multiple: Primary endpoint variable	Advice/Concern regarding endpoint

									Analyses
	Primary analysis								
Free Text	Drop-down	Drop-down	Free Text	Drop-down	Free Text	Free Text	Drop-down	Free Text	Drop-down
Other: Advice/Concern regarding endpoint	Confirmatory testing	Comparisons	Other: Comparisons	Type of analysis	Other: Type of analysis	Analysis Method	Advice/Concern regarding primary analysis	Other: Advice/Concern regarding primary analysis	Use of IA?

Interim analyses				Purpose of interim analyses					
Integer	Drop-down	Drop-down	Free Text	Drop-down	Drop-down	Drop-down	Drop-down	Free Text	Free Text
Number of IA	Use of (i)DMC?	Advice/Concern regarding interim analysis	Other: Advice/Concern regarding interim analysis	Efficacy stop of a treatment arm	Futility stop of a treatment arm	Addition of new treatment arm	Adaptive modification of trial	Which modification?	Other purpose

Multiplicity					
	Reason for multiplicity control over treatment arms				
Drop-down	Drop-down	Drop-down	Drop-down	Drop-down	Free Text
Multiplicity control over treatment arms?	Shared control	Use of multiple treatment arms	Having similar treatment arms	Single claim of efficacy	Other: Reason for multiplicity control

Reason for no multiplicity control over treatment arms					Type 1 error adjustment				
Drop-down	Drop-down	Free Text	Drop-down	Free Text	Drop-down	Drop-down	Drop-down	Drop-down	Drop-down
Only logistical joint platform	Independent claim of efficacy	Other: Reason for no multiplicity control	Advice/Concern regarding multiplicity	Other: Advice/Concern regarding multiplicity	Closed testing	Hierarchical testing	Bonferroni	Dunnett	Graphical approach

		Overall advice			
Free Text	Drop-down	Drop-down	Free Text		
Other method	Not mentioned	Advice/Concern overall study	Other: Advice/Concern overall study	EMA Procedure	Internal: Reviewers comment for discussion or adjudication
